# Editorial: Language and culture in organization and consumer behaviors

**DOI:** 10.3389/fpsyg.2023.1266220

**Published:** 2023-10-13

**Authors:** Xi Li, Tomoki Sekiguchi, Kui Yi, Qingyu Zhang, Luluo Peng, Ligang Zhang

**Affiliations:** ^1^School of Economics and Management, Nanchang Institute of Technology, Nanchang, China; ^2^School of Economics, Kyoto University, Kyoto, Japan; ^3^School of Economics and Management, East China Jiaotong University, Nanchang, China; ^4^College of Management, Shenzhen University, Shenzhen, China; ^5^School of Business Administration, Hunan University, Changsha, China; ^6^School of Journalism, Fudan University, Shanghai, China; ^7^School of Grammar and Law, East China University of Technology, Nanchang, China

**Keywords:** organizational language, organizational culture, consumer behaviors, organizational behavior, cross-cultural development

## 1. Introduction

Language and culture can have a profound effect on organizational strategy, organizational behavior and consumer behavior. In daily life, language is the basic tool for human communication, while culture shapes the values, beliefs and behaviors of individuals (Arno, 2003). In the field of organizational strategies, the differences in language and culture affect companies' decisions and operations in cross-cultural environments (López et al., 2017). Companies with cross-cultural businesses need to focus on the adaptability of organizational strategies in terms of language barriers, cultural differences and communication styles to ensure their effective implementation. In the field of organizational behavior, the selection and application of language in multilingual environments can affect the effectiveness of collaboration and the quality of communication within teams (Lee and Kramer, 2016). Companies need to promote cultural sensitivity and develop cross-cultural communication skills to establish multicultural work environments which will promote teamwork and enhance employee satisfaction. Furthermore, in the field of consumer behavior, language and culture can have a significant impact on consumer needs, preferences and purchasing decisions (Dey et al., 2020; Li et al., 2021; Ye et al., 2023). When marketing and positioning products, companies need to take into account the linguistic and cultural context of target consumers to provide products and services that meet their needs and values. Thus, it is important to understand the impact of language and culture on organizational strategy, organizational behavior, and consumer behavior (Sugita and Takahashi, 2015), and in this regard, our research group examines the impact of language and culture on organizations and consumers from multiple perspectives, including psychology, sociology, and organizational behavior.

Manuscript review is a key quality control step aimed at ensuring that journals publish only high-quality, original academic research. Our research group adheres to the standards of high efficiency and quality in reviewing. According to our past experience, it takes the following processes from the manuscript reception to the paper publication, which usually takes 2–3 months. To begin with, our research group receives the paper and conducts an initial assessment, which is to check whether the paper meets the journal's subject scope and basic requirements, as well as formatting and writing standards, and to make corresponding suggestions for the authors to modify their paper. Next, our research group assigns the assessed papers to at least two external peer reviewers to perform close reviews, evaluating their academic quality, methodology, data analysis, results and conclusions, and to make corresponding comments ask for further revision of the paper. Afterwards, the editorial board evaluates the revised paper to ensure that the authors have adequately responded to the reviewers' comments and made appropriate refinements. After proper modification, the paper is then submitted to the editor-in-chief of the journal for final review. Generally accepted papers are characterized such as high academic quality, great originality, methodological rigor and well-defined conclusions.

There are three general types of manuscript rejection in our research group, which are desk rejection, rejection after initial assessment and rejection after peer review, and the percentages of the three cases in the previous review process respectively are 33%, 56% and 11%. The papers with desk rejection generally have the following two problems: first, the paper is outside the scope. Second, the paper has serious academic problems, such as inappropriate methodology, untrustworthy data, and flawed research design. The above problems may lead to unreliable or non-reproducible conclusions, so an immediate rejection is carried out. Papers rejected after initial assessment generally have the following two problems: first, the paper is insufficiently revised. Second, there are persistent academic problems. The paper fails to solve the academic problems after the initial assessment. There are relatively minor percentage of papers rejected after peer review. The rejection was generally due to the paper still can't meet the journal's requirements after the third round of revision, including flaws in methodology and poor statistical analysis. Our research group will justify the rejection of each paper and give the author detailed feedback to encourage improvement and following high-quality research.

At present, our research group has a total of 25 related papers, concerning the influence of language and culture on organizational behavior, consumer behavior, and their internal mechanisms. From the perspective of culture, they explore the fields such as cultural resources (Zhou et al.), consumer behavior (Zong et al.; Cai and Yu), value co-creation (Yang et al.), tourism (Tuo et al.), social governance (Li et al.). From the perspective of language, they explore the impact of film language (Zeng et al.), language of e-commerce livestream anchors (Ma et al.), visual language (Leng et al.), social media language (Guo et al.) on consumer behavior and learning performance. Above studies will help researchers with same pursuit to better understand the role and function of culture and language in different fields.

In order to better grasp the overall framework and to explore into new research directions and intersections, this paper initially sorts the basic ideas of all published papers and conducts an in-depth analysis of 25 existing papers, using the LDA topic model to reveal the underlying topic structure of the existing research (Blei et al., 2003). First, the researchers pre-processed the text of 25 papers, including removing stop words (such as “the,” “and” and other function words), punctuation marks and numbers. Next, performing stem or lemmatization. The researchers transformed the words into their basic forms to minimize the effect of inflections. And then, the bag-of-words model is formed. The researchers then further construct the topic model and determine the optimal number of topics through the perplexity and coherence score. At last, the topic modeling visualization is guided through the PyLDAvis, and yields the keywords and their weights under each topic (Jelodar et al., 2019).

## 2. Laws between the culture and language of companies and the behavioral performance of consumers

### 2.1. Interesting diversity of topics

For the preliminary analysis of the 25 existing papers in our research group, there are multi-disciplinary and multi-perspective studies of language and culture in both organizational and consumer behavior. From the perspective of language, studies have explored the mechanisms by which vloggers' language affects consumers' purchase intention (Sheng et al.), tactile compensatory effects of visual language on web products (Leng et al.), relationship between dialects and total factor productivity of companies (Xiong and Chen). Studies have validated the impact of an online instructional intervention on college students learning performance of foreign Language during COVID-19 (Xu and Zou), and the dual mediating effect of vloggers' persuasive strategies on consumers' purchase intentions (Sheng et al.). Additionally, there are studies that point out social media marketing and brand equity enabling student behavioral engagement in the higher education (Ruangkanjanases et al.). There are also studies investigating the role of visual language in the online relationships and in constructing images of urban spaces (Liu et al.). From the perspective of culture, some studies have explored the mechanisms between cultural values and consumer behavior (Yang et al.), the relationship between culture and hierarchical medical systems (Tao et al.), and the impact of culture on public services motivation (Duan et al.). Some studies confirmed the resource curse in Chinese culture, revealing that cultural resource endowment could be counterproductive to a certain degree (Zhou et al.). Whereas, studies based on the theory of planned behavior explored the mechanism of consumer purchase intention in traditional culture (Zong et al.), others analyzed the impact of value co-creation on consumer citizenship behavior from a consumer perspective (Yang et al.). There are also studies that have explored the influence of culture in fields such as donation behavior (Wang et al.), movie-induced tourism (Zeng et al.; Lao et al.), and live e-commerce anchors' language appeals (Ma et al.). In addition, there are also studies that connect language and culture at the same time, exploring how they jointly influence consumer behavior and organizational behavior. For instance, the moderating role of cultural differences between on-camera language and linguistic landscapes has been verified, and the empathic mechanism of movie-induced tourism has been explored (Zeng et al.). From the perspective of content creators, there are research analyzed the impact of the co-created cultural and linguistic communities by creators and consumers on the consumer behavior (Ren et al.).

### 2.2. Intersection of Research Topics

**Figure 1:**
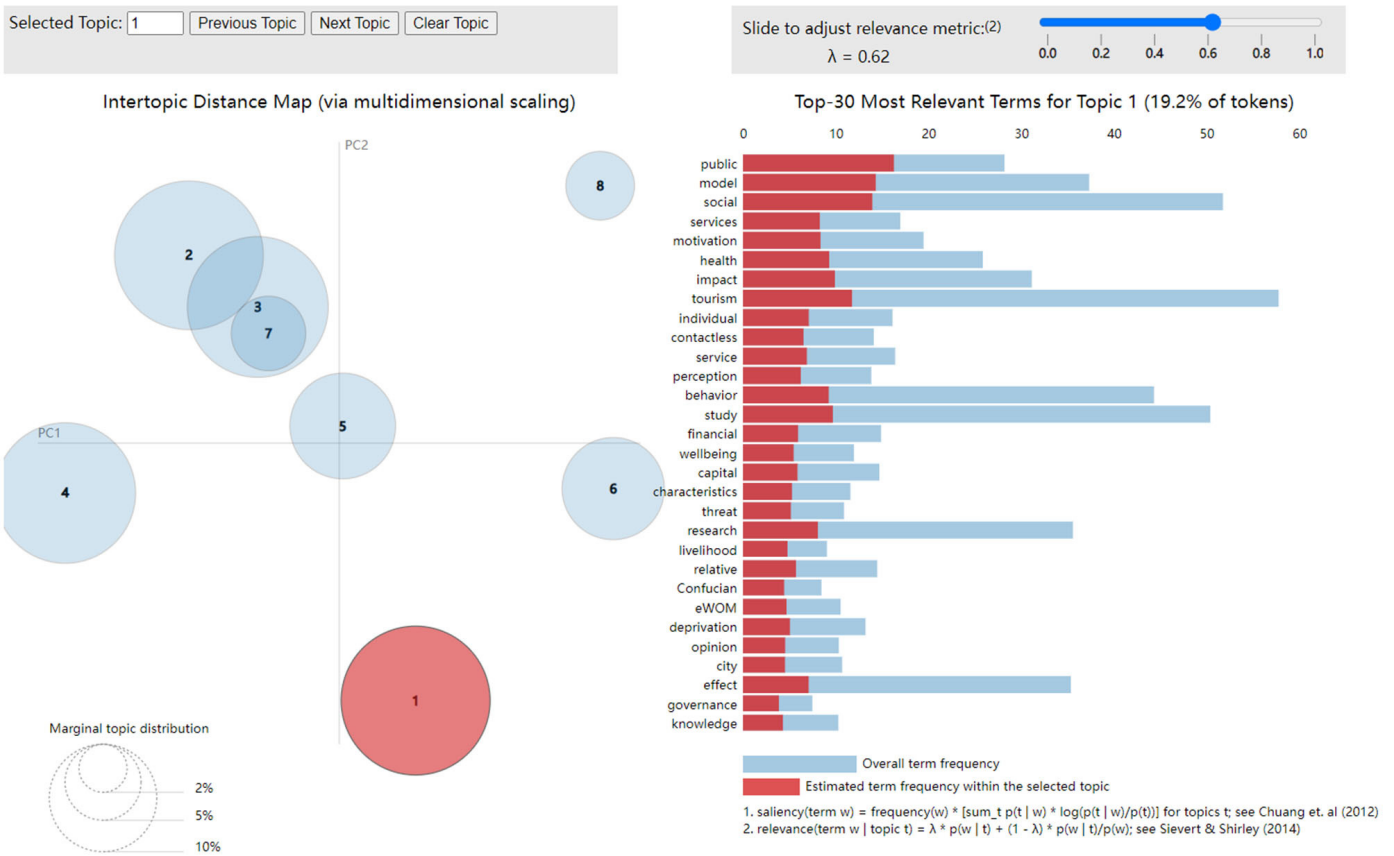
Distribution of LDA topic model.

In order to have a more in-depth understanding of the topic distribution of the existing papers, discover the key topics in the proceedings, mine some new research directions and potential intersections, this paper has employed the LDA topic model to analyze 25 papers, and the results are as follows. First, the perplexity and coherence score were used to judge the optimal number of topics. According to the corresponding curve, it can be seen that the perplexity has the lowest value, and the coherence score has the highest value when the number of topics is around 8 (Hannigan et al., 2019). Therefore, the number of papers related to the topic in this research is determined to be 8. Next, the LDA model is used for visualization, and the results are shown in Figure 1. In the distribution diagram of LDA topic model, one circle represents one topic, and when the number is 8, those circles are basically dispersed and partially intersected, which indicates that the number of topics can cover most of the contents of the existing research texts, and the topic modeling is good (Park et al., 2019). Circles 1–8, respectively correspond to the topics “Culture and Social Interaction,” “Diversity of Cultural Resources,” “Language Culture and Tourism,” “Language and Social Interaction,” “Language and Cultural Interaction,” “Culture and Consumer Experience,” “Culture and Tourism Experience,” and “Language and Education.” The LDA topic model distribution diagram shows the correlation and independence between each topics. Although some of the topics in the distribution diagram overlap to some extent, overall the topics are relatively dispersed in the topic space, which reflects the diversity and broad coverage of the collection of existing studies, with each topic in the diagram representing a different issue or research field (Du et al., 2020).

It can be drawn that the topics in our research group have a distinct intersectional pattern, exploring the mechanisms of linguistic and cultural influences from different fields as follows. First, in the field of marketing, some studies explored the effects of traditional culture and value co-creation on purchase intention, donation behavior and consumer intentions to pay a premium. Second, in the field of tourism and culture, some studies examined from the side of short videos (Qin et al.), movies, tourism public opinion management, and cultural heritage narratives, exploring the role of language and culture in promoting cultural tourism and shaping the spatial imagery of cities. Third, in the field of education, many studies have discussed the impact of online teaching on student performance during COVID-19 and the role of social media marketing in higher education. Fourth, some studies have examined the impact of relative deprivation on health inequalities, and factors influencing behavioral intentions to use contactless financial services in the banking sector, concluding that the impact of language and culture has extended to the health sector (Tao et al.), as well as the financial services sector (Chen et al.).

Consumers can be found in different fields and are accompanied by consumption behaviors, so the influence of language and culture on consumer behavior in existing studies can be summarized in two aspects. On the one hand, language and culture can have an impact on consumers' brand preferences and purchase decisions. Consumers from different linguistic and cultural backgrounds have differences in their demands and preferences for products, brands, and services (Torelli and Stoner, 2019). Understanding and adapting to the linguistic and cultural characteristics of consumers can better meet their needs and boost their purchase intentions. On the other hand, language and culture can work on advertising and marketing. The linguistic and cultural background of the target consumer needs to be taken into account in advertising and marketing to ensure that the information conveyed correctly (Gong et al., 2022). The use of specific language, signs, symbols and cultural elements can be effective in capturing the attention of consumers and creating emotional connections.

In the context of globalization, cross-cultural communication is made more important (Peng and Wu, 2019). In the process of international market expansion, the differences in language and culture will have an impact on business negotiations and customer relations and so on. With the diversity and internationalization of the market, providing multilingual services and localized experiences becomes an important strategy to attract consumers (Zegers and Auron, 2022). Consequently, subsequent research could focus more on the impact of cross-cultural communication, language services and localization on aspects such as organizational or consumer behavior.

## 3. Future research

The general pattern of our research group provides a glimpse of the direction for future research. First, researchers need to examine the impact of language and culture on organizational culture, employee motivation and knowledge sharing to enhance organizational performance and employee satisfaction. Second, researchers need to further study the role of language and culture in social interaction such as education, social governance, political identity and social capital construction in order to promote harmony and development. Third, researchers need to explore the application of language and culture in the field of digital technology, such as artificial intelligence and virtual reality, in order to improve the interactivity and user experience.

The intersection between language and culture and digital technology presents many opportunities and they will also become more prevalent and important in modern society (Lee and Daiute, 2019; Lee et al., 2023; Lyu et al., 2023). On the one hand, with the development of digital technology, the corresponding products and services are launched to the global market, so companies need to consider the user experience from different languages and cultural backgrounds (Guy, 2019). Research on the intrinsic relationship between language and culture and consumers of digital technology can ensure that digital products match the habits, values, and communication styles of consumers from different cultures and provide a better user experience. On the other hand, the development of digital technologies has provided new means for the preservation and presentation of cultural heritage, among other things. Virtual reality and augmented reality technologies provide users with immersive experiences, in which linguistic and cultural elements are incorporated to enable them to experience environments, art and literature produced by different language and culture, in a more intuitive and immersive way (Yeh et al., 2020). Thus, studying the mechanism of the influence of linguistic and cultural elements on the virtual experience, and integrating the two can enhance the user experience, as well as allow for a more targeted preservation and wider dissemination of culture. Not only that, social media provides a platform for people to communicate and interact globally, and language and culture play as a tool for users to express their opinions and share their culture (Yeh and Swinehart, 2020). It can be seen that the intersection of language and culture and social platforms can bring new opportunities for cross-cultural communication and cooperation (Abubaker et al., 2020). The intersection between language and culture and digital technology brings not only globalization and cross-cultural communication, but also new opportunities and challenges (Martínez-Caro et al., 2020). Fostering the intersection can fully exploit the potential of both and promote cross-language and cross-cultural digital development.

## Author contributions

KY: Conceptualization, Data curation, Project administration, Software, Writing—original draft. XL: Investigation, Methodology, Resources, Supervision, Writing—original draft, Writing—review and editing. TS: Data curation, Formal analysis, Supervision, Writing—original draft. QZ: Formal analysis, Funding acquisition, Resources, Validation, Visualization, Writing—review and editing. LP: Data curation, Methodology, Project administration, Supervision, Visualization, Writing—review and editing. LZ: Conceptualization, Investigation, Methodology, Supervision, Writing—review and editing.
